# Interacting stressors and the potential for adaptation in a changing world: responses of populations and individuals

**DOI:** 10.1098/rsos.161057

**Published:** 2017-06-21

**Authors:** Gareth R. Hopkins, Susannah S. French, Edmund D. Brodie

**Affiliations:** Department of Biology and the Ecology Center, Utah State University, 5305 Old Main Hill, Logan, UT 84322, USA

**Keywords:** amphibian, newt, salinity, stress, temperature, variation

## Abstract

To accurately predict the impact of environmental change, it is necessary to assay effects of key interacting stressors on vulnerable organisms, and the potential resiliency of their populations. Yet, for the most part, these critical data are missing. We examined the effects of two common abiotic stressors predicted to interact with climate change, salinity and temperature, on the embryonic survival and development of a model freshwater vertebrate, the rough-skinned newt (*Taricha granulosa*) from different populations. We found that salinity and temperature significantly interacted to affect newt embryonic survival and development, with the negative effects of salinity most pronounced at temperature extremes. We also found significant variation among, and especially within, populations, with different females varying in the performance of their eggs at different salinity–temperature combinations, possibly providing the raw material for future natural selection. Our results highlight the complex nature of predicting responses to climate change in space and time, and provide critical data towards that aim.

## 1. Introduction

Few, if any, environments are truly stable and free of stressors that disrupt homeostasis and threaten survival [1]. Additionally, it is widely recognized that organisms rarely face a single stressor in isolation in these environments, and there is the potential for multiple stressors to interact in complex ways (e.g. [2–6]). Climate change arguably increases this potential [2,7,8]. To accurately predict the consequences of climate change on populations and species, it is critical to assay the responses of sensitive organisms to key environmental stressors, and yet, this information is lacking for most organisms [9]. Understanding how species, populations and individuals persist, develop, reproduce and regulate physiological processes in the face of interacting stressors is therefore a key priority area of research for biologists.

Temperature and salinity have long been recognized as two of the most important abiotic factors affecting the lives of aquatic organisms, and have the potential to interact significantly with climate change [8,10–12]. Temperature and salinity have, individually, been shown to influence demography [13–15], survival, growth, development, reproduction (e.g. [16–19]), mobility [20] and even predator–prey interactions [21] of aquatic organisms. Further, temperature and salinity can interact in critical ways, with one stressor influencing the potency of the other (e.g. [11,16,22,23]).

While the interactions of salinity and temperature have been well studied in marine and estuarine organisms, much less work has been conducted on freshwater taxa (but see [24–32] examining each stressor in isolation). This is in spite of the fact that elevated salinity in freshwater habitats due to road de-icing salt application (e.g. [33,34]), changing land-use patterns (e.g. [35,36]) and coastal salt-water intrusion from rising sea levels (e.g. [37,38]) is of increasing conservation concern [39]. With climate change-associated alteration of weather patterns, global temperatures and sea-level rise, the likelihood of temperature and salinity interacting to influence the survival of organisms in freshwater environments only increases [8,10–12]. The interaction of salinity and temperature on metabolism and nitrogen exchange has been investigated in the model freshwater fishes *Gambusia affinis* and *Danio rerio* [40], but the majority of studies have been conducted on euryhaline fishes such as *Cyprinodon macularius* [41], *Menidia beryllina* [42] and *Fundulus* species (principally *Fundulus heteroclitus*) [43–45]. By contrast, much less work has been conducted on freshwater organisms perceived to be especially sensitive to salinity stress. Owing to their permeable skin and eggs, requirement of aquatic systems for reproduction, and ectothermic nature, amphibians have long been considered ideal sensitive freshwater models with which to examine the effects of salinity and temperature (albeit generally with each stressor presented in isolation) (e.g. [31,32,46–52]). Peabody & Brodie [53] examined the effects of both salinity and temperature on embryonic development and vertebral number of the salamander *Ambystoma maculatum*, and an early study on the tailed frog *Ascaphus truei* indicated that salinity and temperature in combination were much more lethal to adults and larvae than each stressor alone [54]. More recently, salinity and temperature were found to interact to affect larval survival and development in two species of European toads *Epidalea calamita* and *Bufo viridis* [55,56]. To the best of our knowledge, these studies represent the only examination of the interaction of salinity and temperature in amphibians.

Intriguingly, these studies on European toads [55,56] also highlight that different populations of the same species may vary in their resiliency to stressor combinations, which could have important implications for conservation efforts [56]. Populations may vary in their resiliency due to different evolutionary histories of exposure to the stressors [57–60], and the importance of evolutionary processes, including local adaptation, in this context is beginning to be realized [7,55,56,59,61]. While population-level responses give insight to the current mean response of the population, individual-level responses (i.e. within populations) allow insights into the potential for populations to adapt to future changes in stressor exposure, as intrapopulation variation is critical for natural selection [9,46,62,63]. Despite its potential importance for conservation [9,64], the variability of responses to multiple stressors among, and especially within, populations remains understudied [9].

**Figure 1.**
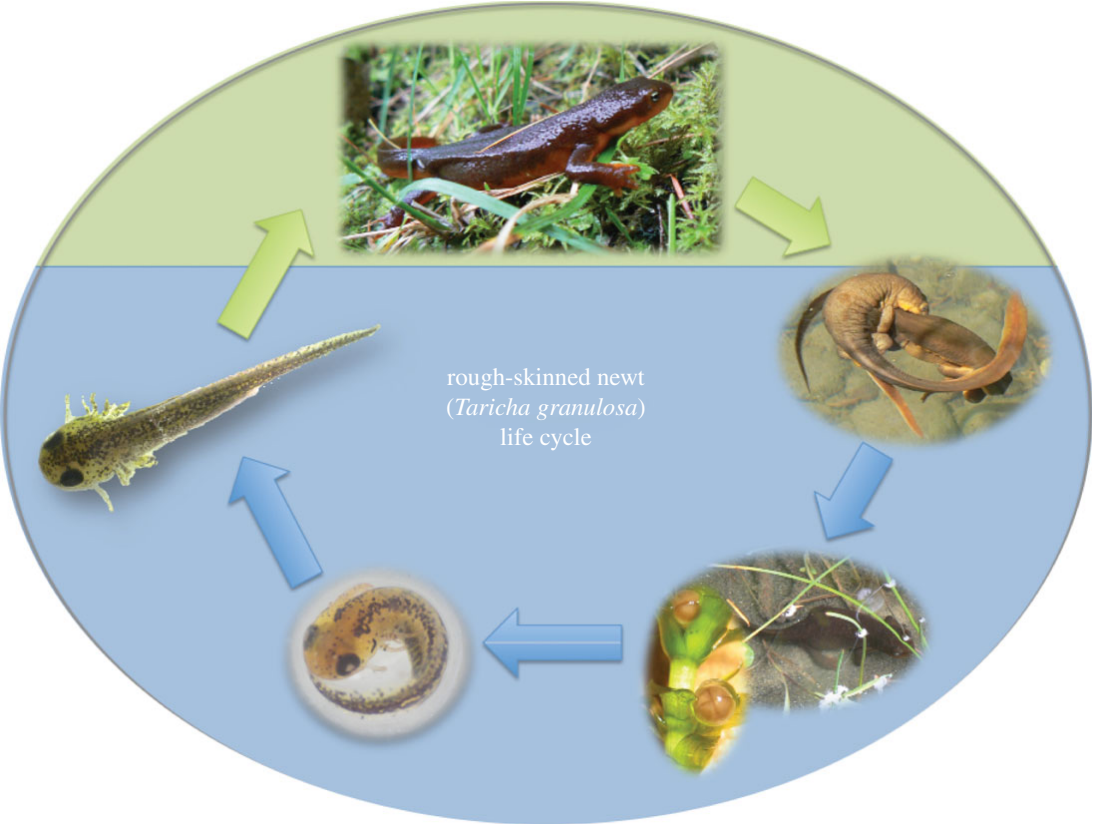
Life cycle of the rough-skinned newt (*T. granulosa*). Adults live terrestrially (green background), but return to ponds and streams (blue background) to breed every spring. Mating occurs in the water, followed by the deposition of fertilized eggs on aquatic littoral vegetation. Eggs develop and hatch over the next month, and gilled aquatic larvae grow and develop over the next several months before metamorphosing and returning to land. Photos by G.R.H., B. Gall and A. Spence. Adapted, with permission, from [65].

We examined the interacting effects of temperature and salinity stress on the embryonic survival and development of a North American amphibian, the rough-skinned newt (*Taricha granulosa* Skilton; Caudata: Salamandridae; figure 1), from populations that varied in their evolutionary history of exposure to salinity and temperature regimes. Rough-skinned newts inhabit a variety of lentic and lotic habitats [66] that naturally vary in both temperature and salinity ([67]; G.R.H. and Z. M. Hopkins 2012–2014, unpublished data), making them excellent models to examine the interaction of these stressors on freshwater organisms. We examined both inter- and intra-population variation in responses to salinity and temperature, to investigate the potential for freshwater organisms to adapt to combinations of these stressors. We hypothesized that the effects of each stressor would be highly dependent on the levels of the other, that newts from different populations would vary in their responses according to their differing evolutionary history with each stressor and that there would be high individual variation in responses within each population, which may allow for future adaptation.

## 2. Material and methods

### 2.1. Organism

Rough-skinned newts (*T. granulosa*) (figure 1) are caudate amphibians commonly found living in forests, streams and ponds along the Pacific coast of North America, from southern California to Alaska [66]. Newts spend most of the year in the forest but return to breed in aquatic systems every spring, where they mate in the water and individually lay jelly-covered eggs among littoral vegetation. Eggs take several weeks to months to develop (depending on the temperature), with gilled aquatic larvae hatching and living in the water for several months before metamorphosing as terrestrial juveniles (figure 1).

### 2.2. Populations and field collection

We collected gravid female rough-skinned newts (11–12 per population) by dip-net, minnow trap and hand from two locations in Oregon, USA, 14–21 May 2013: a collection of human-made freshwater ponds (Soap Creek ponds, Benton County—hereafter ‘Soap Creek—SC’, approx. 62 km inland from the Pacific Ocean; 44°40.236′ N, 123°16.613′ W; see [46,62] for more information on this habitat), and a coastal tidal stream, Hunter Creek (Curry County, 42°23.306′ N, 124°25.228′ W; see [67,68] for more details on this habitat). At Hunter Creek, three ‘populations’ were sampled: newts found in the tidal area of the stream, up to 500 m from the Pacific Ocean (hereafter ‘Hunter Tidal—HT’), newts found approximately 3.5 km upstream from the tidal area in a freshwater, non-tidal area of the stream (hereafter ‘Hunter Fresh—HF’) and newts found a further approximately 3.5 km upstream from that location (7.5 km from the ocean, hereafter ‘Hunter Upper Fresh—HUF’). While newts occur throughout Hunter Creek, for the purposes of this paper, we are referring to these three locations as ‘populations’, as related species of *Taricha* generally do not disperse more than 1.3 km between breeding sites [69].

Populations were chosen to represent potentially different histories of exposure to salt (decreasing history of exposure travelling inland), as well as possibly different thermal regimes (pond versus stream). Salinity and temperature in all locations were measured using a handheld YSI EC300 multimeter (YSI, Yellow Springs, OH, USA) on multiple days and locations within the habitats, and at both high and low tide (for HT). Water temperature varied considerably at all sites, depending on weather, shade and depth, and ranged from 8 to 21°C at Hunter Creek (mean temperature at HT = 15.7°C, HF = 14°C, HUF = 13.6°C), and 12 to 23°C at the Soap Creek Ponds (mean = 16.6°C) during the newt breeding season (May). Salinity was consistently 0.0–0.1 ppt in all locations, with the exception of HT, which experienced intermittently elevated salinity from the ocean. Salinity at HT varied depending on depth, distance from the ocean, tides and microhabitat, and ranged between 0.1 and 1.4 ppt. While we did not measure highly elevated salinity in HT, this habitat is subjected to occasional storm events that may raise salinities to levels much greater than those measured (see [67,68] for more details on this habitat). By contrast, newts at the inland Soap Creek ponds are never subjected to increased salinity, as they are located over 300 m from the nearest minor road, which is not treated with road de-icing salts [46].

### 2.3. Female newt husbandry and oviposition

Female newts were transported back to the laboratory and housed individually in non-leaching plastic containers (35 × 20 × 13 cm) with 2.0 l of aged, chilled tap water filtered to remove chlorine (salinity = 0.2 ppt) and a styrofoam perch, in a temperature-controlled room at 14°C, with a 12 L : 12 D cycle. After 3 days of acclimation, newts were injected with 10 μl luteinizing hormone-releasing hormone ([des-Gly^10^, _D_-His(Bzl)^6^] LH-RH ethylamide; Sigma No. L2761; Sigma Aldrich, St Louis, MO, USA) to induce oviposition, perches were removed and replaced with submerged polyester fibre to serve as an oviposition substrate. Newts began laying eggs within 24 h. Individual eggs were removed from the substrate and randomly assigned to a temperature–salinity treatment within 24 h of oviposition.

### 2.4. Salinity and temperature treatments

Salinity treatment solutions were made by mixing laboratory grade NaCl (Mallinckrodt Baker, Inc., Paris, USA) and filtered tap water to a concentration of either 2.0 or 5.0 ppt. These salinities were chosen as concentrations that could be found in roadside environments impacted by road de-icing salts [70], as well as estuarine environments [71], that can seriously affect newt survival, development and physiology [46,67]. The filtered tap water (0.2 ppt) was used as a freshwater control. Temperature-controlled rooms were set to 7, 14 or 21°C, representative of the range of temperatures that newts may experience during the breeding season in the wild, and which are known to significantly influence embryonic and larval growth and development in this species [52].

### 2.5. Experimental procedure

Within 24 h of oviposition, eggs (mean ± s.d. egg diameter = 2.67 ± 0.27 mm, *N* = 481) were removed from their mother's container and placed singly in 3.5 cm diameter, 1 cm deep lidded round plastic Petri dishes with 4 ml of randomly assigned salt or control solution. This Petri dish was then randomly assigned to one of the three temperatures. This procedure was continued until all females from all populations had approximately 10 eggs (replicates) assigned to each temperature–salt combination. Eggs were reared based on the methods of Hopkins *et al.* [46] and were checked daily for mortality or hatching. Water levels in Petri dishes were checked daily for evaporation and replaced with small amounts of distilled water to maintain constant salinity. Upon hatching, the total length of larvae was measured using an ocular micrometer attached to an Olympus stereomicroscope, and developmental stage [72] determined. Rough-skinned newts typically hatch at Harrison stages 39–42, depending on environmental conditions, population of origin and maternal identity (figure 4*c* in Results). These developmental stages are characterized by the full development of gills and the beginning development of the fore-limb bud (see [62,72] for further details). The presence of any developmental deformities was recorded using the criteria of Hopkins *et al*. [73].

### 2.6. Statistical analyses

Egg survival (%), time to hatching, length at hatching, developmental stage at hatching and the presence of developmental deformities (%) were analysed using generalized linear mixed model ANOVAs with the Identity link function and restricted maximum-likelihood estimation of random effects using PROC GLIMMIX in SAS^®^ v. 9.4. Significance was set at *α* = 0.05. The fixed effects in these models were: salinity, temperature, population and the interactions of all of these factors. To investigate the variation among females within populations, female identity was incorporated as a random effect, crossed with temperature and salinity, and nested within population. Because of very high mortality at 5 ppt, the sample size of surviving embryos was greatly reduced to examine sublethal effects. These effects (time to hatching, length at hatching, developmental stage at hatching and the presence of developmental deformities) were therefore analysed both including and excluding survivors at 5 ppt. We present the full model results in the main text and provide the models excluding 5 ppt in electronic supplementary material, table S1.

## 3. Results

### 3.1. Egg survival

**Figure 2.**
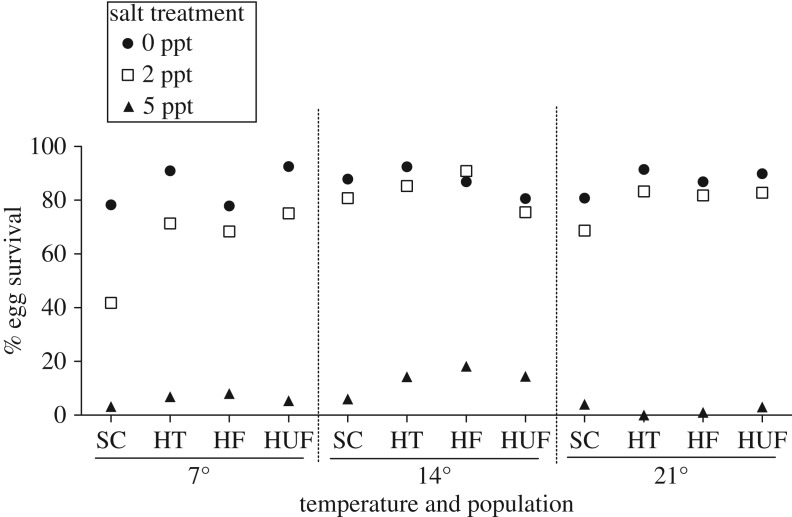
Per cent survival in salinity (0, 2, 5 ppt) and temperature (7, 14, 21°C) treatments of eggs from rough-skinned newts (*T. granulosa*) from four different populations.

**Figure 3.**
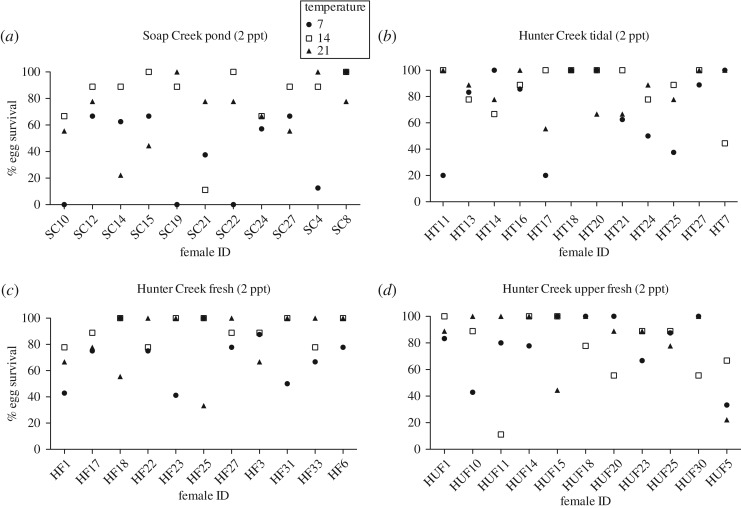
Interfamily variation in per cent survival of eggs from 11 to 12 different female newts (*T. granulosa*) from each of four different populations in 2 ppt salinity in different temperature treatments (figures showing survival in other salinity treatments can be found in the electronic supplemental material).

Salinity, temperature and the interaction of salinity and temperature, all significantly affected egg survival (table 1*a*). Increased salinity generally led to decreased survival, with extremely low survival at 5 ppt (less than 10%, except for the Hunter Creek populations at 14°C) (figure 2). The negative effects of salinity were greatest at 7°C and less at 14°C (figure 2, table 1*a*). Soap Creek newts in general had lower average survival than Hunter Creek newts (significant effect of population, table 1*a*, Tukey–Kramer post hoc multiple comparisons among populations *p* < 0.05), particularly at increased salinities (significant interaction of population and salinity, table 1*a* and figure 2). There was not a significant three-way interaction of population with salinity and temperature (table 1*a*). Rather, within populations, individual females varied in the survival of their offspring in different salinity and temperature combinations (table 1*b* and figure 3; electronic supplementary material, figures S1 and S2). Intrapopulation variation among females was generally high among all populations and in all treatments (except for 5 ppt; table 2). Different females in each population appeared to have different specific salinity–temperature combinations best suited for the survival of her eggs (variance estimate for female × salinity × temperature, table 2 and figure 3; electronic supplementary material, figures S1 and S2). For example, female SC22 experienced 100% survival of her offspring in 2 ppt in 14°C, but 0% survival in the same salt concentration at 7°C (figure 3*a*), while female HUF11 experienced almost exactly the opposite pattern (figure 3*d*).

**Figure 4.**
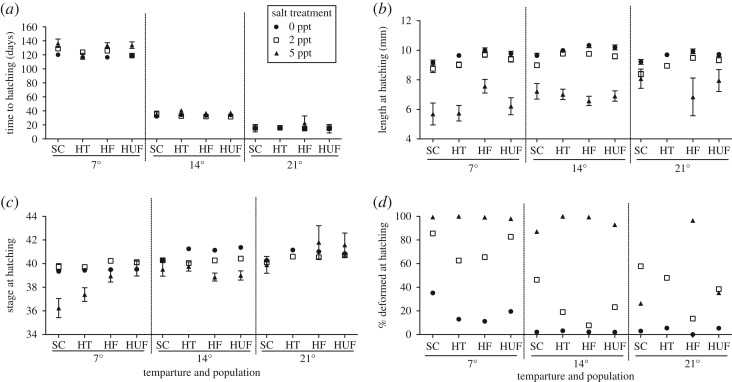
Sublethal effects of salinity and temperature embryonic developmental stress on hatchling newts (*T. granulosa*) from four different populations. Means ± s.e. (*a*) Time to hatching (days), (*b*) length at hatching (mm) and (*c*) developmental stage at hatching (Harrison stages) [72]. (*d*) Percentage of offspring born with a developmental deformity.

**Table 1.** Effects of salinity, temperature, population and their interactions on newt (*T. granulosa*) embryonic survival. (*a*) Main effects, (*b*) intrapopulation variance among females in offspring survival (random effects). Asterisks highlight statistical significance.

(*a*) fixed effects	*F*	d.f.	*p*-value
salinity	1142.78	2,82	<0.0001*
temperature	5.07	2,82	0.008*
population	4.27	3,41	0.010*
salinity × temperature	5.33	4,164	0.0005*
population × temperature	0.98	6,82	0.45
population × salinity	2.40	6,82	0.035*
population × salinity × temperature	1.27	12,164	0.24
**(*b*) random effects (nested within population)**	**variance**		
female	−0.001		
female × salinity	−0.001		
female × temperature	0.010		
female × salinity × temperature	0.014		

*p < 0.05.

**Table 2.** Intrapopulation variation (minimum, maximum, mean) in egg survival (%) among females (*N* numbers) from four newt (*T. granulosa*) populations under different salinity–temperature (°C) treatment combinations.

treatment	population											
	SC (*N* = 11)			HT (*N* = 12)			HF (*N* = 11)			HUF (*N* = 11)		
temperature	min	max	mean	min	max	mean	min	max	mean	min	max	mean
0 ppt												
7	11.11	100	78.79	57.14	100	90.76	55.56	100	79.81	75.00	100	92.42
14	55.56	100	87.88	87.85	100	93.52	66.67	100	86.87	44.44	100	86.74
21	55.56	100	80.81	77.78	100	91.67	22.22	100	86.87	66.67	100	89.90
2 ppt												
7	0.00	100	42.70	20.00	100	70.66	41.18	100	72.16	33.33	100	79.22
14	11.11	100	80.81	44.44	100	31.46	77.78	100	90.91	11.11	100	75.76
21	22.22	100	68.69	55.56	100	9.082	33.33	100	81.82	22.22	100	82.83
5 ppt												
7	0	11.11	3.03	0	33.33	7.13	0	25.00	8.37	0	25.00	5.59
14	0	22.22	6.06	0	55.56	13.89	0	44.44	18.18	0	33.33	14.43
21	0	33.33	4.04	0	0	0	0	11.11	1.01	0	22.22	4.04

### 3.2. Time to hatching

Temperature had the greatest effect on time to hatching (table 3*a*; electronic supplementary material, table S1*a*), with eggs hatching in approximately 16 days at 21°C, 33 days at 14°C and 124 days at 7°C (figure 4*a*; electronic supplementary material, table S2). Salinity significantly interacted with temperature (table 3*a*; electronic supplementary material, table S1*a*), such that the effects of increased salinity delaying hatching were most significant at 7°C (figure 3*a*). Population played little role in affecting time to hatching (table 3*a*; electronic supplementary material, table S1*a*), although there was large intrapopulation variation in the effects of salinity × temperature interactions on hatching timing (variance estimate of female × temperature × salinity, table 3*a*; electronic supplementary material, table S2 and figures S3–S5).

**Table 3.** Sublethal effects of salinity, temperature, population and their interactions on newt (*T. granulosa*) embryonic growth and development. Main effects as well as the variance among females within populations (random effects) are presented. Results for statistical models excluding individuals who survived exposure to 5 ppt (due to very low survival at 5 ppt—see Results) are found in electronic supplementary material, table S1. Asterisks highlight statistical significance.

	(*a*) time to hatching			(*b*) length at hatching			(*c*) stage at hatching			(*d*) deformities at hatching		
fixed effects	*F*	d.f.	*p*-value	*F*	d.f.	*p*-value	*F*	d.f.	*p*-value	*F*	d.f.	*p*-value
salinity	7.43	2,65	0.0012*	95.65	2,65	<0.0001*	9.99	2,65	0.0002*	21.47	2,65	<0.0001*
temperature	2656.15	2,82	<0.0001*	5.88	2,82	0.0041*	21.81	2,82	<0.0001*	1.15	2,82	0.32
population	1.37	3,41	0.26	2.63	3,41	0.06	5.06	3,41	0.0045*	2.38	3,41	0.08
salinity × temperature	5.49	4,91	0.0005*	1.44	4,90	0.23	10.27	4,89	<0.0001*	10.61	4,90	<0.0001*
population × temperature	1.95	6,82	0.08	2.10	6,82	0.06	1.55	6,82	0.17	8.99	6,82	<0.0001*
population × salinity	0.83	6,82	0.55	0.59	6,65	0.74	2.02	6,65	0.08	2.10	6,65	0.06
population × salinity × temperature	0.86	11,91	0.58	1.27	11,90	0.25	1.46	11,89	0.16	4.85	11,90	<0.0001*
**random effects (nested within population)**		**variance**			**variance**			**variance**			**variance**	
female		1.07			0.05			0.04			−0.002	
female × salinity		−0.27			0.05			0.02			0.14	
female × temperature		2.53			−0.008			0.01			−0.001	
female × salinity × temperature		41.44			0.041			0.16			−0.08	

*p < 0.05.

### 3.3. Length and developmental stage at hatching

Salinity and temperature both influenced length at hatching and developmental stage at hatching (table 3*b,c*; electronic supplementary material, table S1*b,c*), with salinity and temperature interacting to affect stage (table 3*c*; electronic supplementary material, table S1*c*), but not length (table 3*b*; electronic supplementary material, table S1*b*) at hatching. Embryos reared in saline solutions hatched significantly smaller (figure 4*b*) and less developed (figure 4*c*) than those in the control. Effects on development were most dramatically seen at 7°C and 5 ppt (figure 4*c*), and Soap Creek animals were consistently less developed (though not smaller) than animals from any of the Hunter Creek populations (table 3*b,c*, Tukey–Kramer post hoc multiple comparisons among populations *p* < 0.05; figure 4*b,c*). There was a marginal (population × salinity: *p* = 0.08) trend of the eggs of Soap Creek females hatching especially less developed in 5 ppt (figure 4*c*). Females within populations varied in both length (table 3*b*; electronic supplementary material, table S3 and figures S6–S8) and developmental stage (table 3*c*; electronic supplementary material, table S4 and figures S9–S11) of their offspring at hatching under different salinity × temperature combinations.

### 3.4. Developmental deformities

The interactions of salinity, temperature and population all significantly affected the percentage of embryos hatching deformed (table 3*d*; electronic supplementary material, table S1*d*). Increasing salinity leads to increased rates of deformities, with up to 100% of hatchlings being deformed at 5 ppt (figure 4*d*). This pattern was most extreme at 7°C, and dampened significantly at 14°C, with Soap Creek pond animals generally more prone to deformities than Hunter Creek animals in increased salinity (although HUF also tended to have more deformities than HF at various salinities; Tukey–Kramer post hoc multiple comparisons among populations *p* < 0.05). This pattern of SC newts suffering from deformities was especially obvious at 7°C, where over 30% of SC embryos hatched deformed, even in the freshwater control (significant effect of population × temperature, table 3*d*; figure 4*d*). In addition, while the effects of salinity (especially 2 ppt) on deformities were dampened significantly at 14°C compared with 7°C, over 40% of SC hatchlings in 2 ppt and 14°C still hatched deformed (far higher than any of the Hunter Creek populations; significant effect of population × salinity × temperature, table 3*d* and figure 4*d*). Intrapopulation variation in the presence of deformities was low across salinity and temperature combinations, but higher when examining the interacting effects of salinity and female alone (i.e. averaged across temperatures; table 3*d*; electronic supplementary material, table S5 and figures S12–S14). Developmental deformities ranged from abnormal spinal bending to reduced gill or limb development, and abdominal cysts/oedemas (i.e. as seen in [73]).

## 4. Discussion

Salinity and temperature strongly interacted to affect the survival and development of newt embryos in this study, with salinity generally having the greatest effect at temperature extremes. We also detected variation in responses both between and within populations, with different females in each population having different temperature and salinity combinations best suited to the survival and development of their eggs. These results highlight the complex, interacting nature of common abiotic stressors facing organisms, and provide important information for the potential resiliency of populations.

Increased salinity led to decreased survival, increased time to hatching, decreased length and developmental stage at hatching, and a high percentage of developmental deformities, as seen before for amphibians (e.g. [26,28,46,73–75]) and other freshwater organisms (e.g. [25,29,76]). Survival at 5 ppt salinity was especially low (less than 20% across temperatures and populations, and over 90% of survivors being small and deformed), perhaps representing an upper limit of salinity tolerance of newt eggs. While these results were not unexpected due to the high permeability of amphibian eggs, and their lack of effective osmoregulatory mechanisms [77], we found that the effects of salinity were strongly dependent on temperature, with most lethal and sublethal salinity effects being strongest at temperature extremes (7 and 21°C). Even if eggs escaped mortality at these stressor combinations, hatching smaller, less well developed and more deformed has important fitness consequences [78,79]. Similar synergistic patterns of combined salinity and temperature stress have also been shown in fish (e.g. [41,44]) and anuran tadpoles [54–56], where animals were able to tolerate some degree of temperature or salinity stress in isolation, but not in interaction. The toxicity of other stressors, such as pesticides [42,80], polychlorinated biphenyls [8] and dissolved oxygen [41], has also been shown to be amplified at marginal temperatures and/or salinities.

Although relatively little work has been conducted on freshwater organisms, salinity and temperature have repeatedly been shown to interact to affect survival, growth and development of marine and estuarine fishes (e.g. [17,22,81]) and invertebrates (e.g. [12,16,82,83]), with the most favourable conditions for survival and development often found at median temperatures [16,17,22,82]. Indeed, our results, in concordance with these studies, appear to support the general assertion that the interaction of environmental stressors is most impactful at the marginal limits of tolerance of a species to any given stressor [12,43,83], as organisms may be more vulnerable to further stressors when already stressed. As temperature extremes continue to become the norm with climate change, the toxicity of other, interacting, stressors to aquatic organisms, both marine and freshwater, may therefore also increase.

The exact optima of salinity and temperature for marine organisms often varies substantially for different populations or closely related species (e.g. [16,45,58,84,85]). Similar patterns have been found for different populations of the euryhaline European toads *E. calamita* [55] and *B. viridis* [56] (but not for the frog *A. truei* [54]), where the interaction of salinity and temperature impacted some populations more than others. We found in general that population was often a significant predictor of survival and development in our study, with the three Hunter Creek populations grouping together in their responses compared with the Soap Creek pond newts. Soap Creek newts had particularly high egg mortality in salt solutions, and high rates of developmental deformities in 7°C and 2 ppt compared with Hunter Creek newts. Soap Creek pond newts are found furthest from the ocean and are not naturally or anthropogenically exposed to salinity (see [46]). In addition, newts from Soap Creek pond very rarely experience water temperatures as cold as 7°C (G.R.H. and E.D.B. 2012–2104, unpublished data), while newts in Hunter Creek experience a wide range of temperatures ranging from 7 to 21°C, largely dependent on microhabitat depth, flow-rate and weather (Z. M. Hopkins and G.R.H. 2012–2104, unpublished data). Indeed, lentic habitats in general typically experience more stable (and warmer) temperatures than those in lotic habitats. Thus, thermal stress at this temperature may synergize with salinity stress in particular for this lentic population. This combined lack of history with low temperatures and even moderate salinities may thus help explain the relatively high mortality and high occurrence of sublethal effects, the embryos of these animals experienced on average at 2 ppt and 7°C relative to animals from coastal, lotic, Hunter Creek. Interestingly, the embryos of animals in the tidal environment of Hunter Creek (HT) were generally no more saline tolerant than other upstream populations, contrary to our hypothesis and recent results on adult physiology of newts from HT versus HF populations [67]. This may be due to the fact that amphibian eggs cannot osmoregulate as effectively as later life stages [77,78], and so subtle differences among closely grouped populations in any one area (i.e. within Hunter Creek) do not appear at the egg stage. Our ability to extrapolate these findings more generally is hampered by only having one lentic, inland population to compare with several lotic populations from the same coastal stream. Further work is needed sampling across a wider variety of populations in different habitat types in order to gather the evidence needed to fully explain the population-level variation in salinity × temperature tolerance we observe.

While we did not always find significant differences among populations for most traits examined in the interaction of temperature and salinity, we did find a consistent pattern of variation in the responses of different females' eggs within populations to different salinity × temperature combinations, which provides important information on the capacity for the population to evolve in the future [9]. Within any given population, it appears that the eggs of some females persist best in certain salinities at low temperatures, while others do much better at higher temperatures, and the eggs of other females all react similarly to temperature. Although almost all individuals do poorly at 5 ppt, (except one HT female in 14°C, table 2; electronic supplementary material, figure S2), at least at lower salinities, at any given temperature combination, there are individuals within populations whose offspring will survive significantly better than others, and thus shift the mean response value of the population in response to a selection event. If this variation is heritable (which has yet to be definitively determined), then the population can evolve, and evolutionary rescue [86] may occur in future climate change scenarios for this species. It will be important, however, for further work to compare a larger sample size of females from each population and control for other possible sources of variance before definitive interpretations of population adaptability are made. In addition, the importance of this adaptive possibility for conservation must be balanced with the dangers of bottlenecks and inbreeding depression inherent with reduced population sizes resulting from selection events [75,86]. The severity of selection will also be dependent on the severity of the stressor applied (i.e. there may be more potential for population persistence in response to 2 versus 5 ppt salinity), and it is possible that simultaneous selection from multiple stressors may impact the ability for adaptation [55]. Despite these limitations, our results, combined with those of previous studies [46,62], clearly show an intriguing pattern of strong differences in the survival of individual females' offspring in response to salinity and temperature stress, which have the potential to be important for adaptation. While responses of populations and individuals to multiple environmental stressors are clearly complex, taking into account intrapopulation variation in response to multiple, interacting stressors has the potential to provide important insights into the abilities of populations and species to persist in the future [9].

By examining the effects of two of the most common abiotic stressors predicted to interact with climate change on a model freshwater organism, our study has emphasized the importance, as well as complexities therein, of predicting responses of vulnerable organisms to environmental change. Such complexities will undoubtedly only increase in a more natural setting, where additional stressors will also interact, and be applied in unpredictable ways that are difficult to mimic in the laboratory. Taking into account the interacting nature of stressors, in both the laboratory and the field, as well as the variation in responses of organisms both between and within populations, will allow us better predictive power in striving to understand the ability of organisms to survive, develop and reproduce in increasingly stressful environments, now and in the future.

## Supplementary Material

Alternate analysis of sublethal effects and description of intrapopulation variation in responses

## Supplementary Material

Data

## Data accessibility

Original data for this experiment are found in the electronic supplementary material.
