# Supplementary material for: Interacting stressors and the potential for adaptation in a changing world: responses of populations and individuals
Source: R Soc Open Sci. 2017 Jun 21;4(6):161057. doi: 10.1098/rsos.161057 (PMC5493904; doi:10.1098/rsos.161057)
Supplement: Alternate analysis of sublethal effects and description of intrapopulation variation in responses [file rsos161057supp1.pdf]

Supplementary Material – Hopkins et al. 2017. Royal Society Open Science

Table S1. Sublethal effects of salinity, temperature, population, and their interactions on newt embryonic growth and development, both including and excluding individuals who survived exposure to 5ppt (due to very low survival at 5ppt - see Results). Asterisks (*) indicate significant differences (*p*<0.05).

A. Time to Hatching		Full Model			Excluding 5ppt	
Fixed Effects	F	df	*p*	F	df	*p*
Salinity	7.43	2,65	0.0012*	11.65	1,41	0.0015*
Temperature	2656.15	2,82	<0.0001*	5467.41	2,79	<0.0001*
Population	1.37	3,41	0.26	0.70	3,41	0.56
Salinity x Temperature	5.49	4,91	0.0005*	13.78	2,79	<0.0001*
Population x Temperature	1.95	6,82	0.08	0.54	6,79	0.78
Population x Salinity	0.83	6,82	0.55	0.32	3,41	0.81
Population x Salinity x Temperature	0.86	11,91	0.58	0.32	6,79	0.92
Random Effects (nested within Population)	Variance			Variance		
Female	1.07			1.52		
Female x Salinity	-0.27			0.39		
Female x Temperature	2.53			5.69		
Female x Salinity x Temperature	41.44			27.06		
B. Length at Hatching		Full Model			Excluding 5ppt	
Fixed Effects	F	df	*p*	F	df	*p*
Salinity	95.65	2,65	<0.0001*	48.44	1,41	<0.0001*
Temperature	5.88	2,82	0.0041*	21.59	2,79	<0.0001*
Population	2.63	3,41	0.06	13.37	3,41	<0.0001*
Salinity x Temperature	1.44	4,90	0.23	0.61	2,79	0.54
Population x Temperature	2.10	6,82	0.06	0.64	6,79	0.70
Population x Salinity	0.59	6,65	0.74	0.32	3,41	0.81
Population x Salinity x Temperature	1.27	11,90	0.25	0.92	6,79	0.48
Random Effects (nested within Population)	Variance			Variance		
Female	0.05			0.05		
Female x Salinity	0.05			0.03		
Female x Temperature	-0.008			-0.02		

Female x Salinity x Temperature	0.041			0.05		
C. Developmental Stage at Hatching	Full Model			Excluding 5ppt		
Fixed Effects	F	df	*p*	F	df	*p*
Salinity	9.99	2,65	0.0002*	4.10	1,41	0.049*
Temperature	21.81	2,82	<0.0001*	68.94	2,79	<0.0001*
Population	5.06	3,41	0.0045*	4.27	3,41	0.010*
Salinity x Temperature	10.27	4,89	<0.0001*	21.40	2,79	<0.0001*
Population x Temperature	1.55	6,82	0.17	1.24	6,79	0.295
Population x Salinity	2.02	6,65	0.08	1.51	3,41	0.23
Population x Salinity x Temperature	1.46	11,89	0.16	1.14	6,79	0.35
Random Effects (nested within Population)	Variance			Variance		
Female	0.04			0.03		
Female x Salinity	0.02			0.06		
Female x Temperature	0.01			-0.03		
Female x Salinity x Temperature	0.16			0.16		
D. Developmental Deformities at Hatching	Full Model			Excluding 5ppt		
Fixed Effects	F	df	*p*	F	df	*p*
Salinity	21.47	2,65	<0.0001*	226.23	1,41	<0.0001*
Temperature	1.15	2,82	0.32	83.21	2,79	<0.0001*
Population	2.38	3,41	0.08	11.93	3,41	<0.0001*
Salinity x Temperature	10.61	4,90	<0.0001*	19.07	2,79	<0.0001*
Population x Temperature	8.99	6,82	<0.0001*	1.90	6,79	0.09
Population x Salinity	2.10	6,65	0.06	4.30	3,41	0.010*
Population x Salinity x Temperature	4.85	11,90	<0.0001*	2.50	6,79	0.030*
Random Effects (nested within Population)	Variance			Variance		
Female	-0.002			0.000		
Female x Salinity	0.14			0.004		
Female x Temperature	-0.001			0.000		
Female x Salinity x Temperature	-0.08			0.014		

Table S2. Intrapopulation variation (Minimum, Maximum, Mean, and percent Coefficient of Variation (CV)) in time to hatching (mean number of days of 10 eggs for each female) among females (*N* numbers) from four newt populations under different salinity - temperature treatment combinations. Values missing for HT for 5ppt and 21˚C due to 100% egg mortality in this treatment.

Treatment	Population															
A. 0 ppt	SC ($N = 11$)				HT ($N = 12$)				HF ($N = 11$)				HUF ($N = 11$)			
Temperature	Min	Max	Mean	CV %	Min	Max	Mean	CV %	Min	Max	Mean	CV %	Min	Max	Mean	CV %
7	104.6	165	123.6	13.4	100.1	142.3	116.2	9.26	107.8	126.7	116.1	5.76	101.7	141.5	120.6	9.22
14	29.0	35.2	32.31	5.69	30.50	36.67	34.18	4.35	31.71	34.67	33.41	2.92	33.00	35.33	33.53	1.94
21	13.43	15.89	15.06	4.53	15.00	17.11	15.71	4.26	14.22	16.22	15.46	4.22	14.00	16.22	15.35	4.06
B. 2 ppt	($N_{7^\circ}=8$, $N_{14,21^\circ}=11$)				($N = 12$)				($N = 11$)				($N = 11$)			
Temperature	Min	Max	Mean	CV %	Min	Max	Mean	CV %	Min	Max	Mean	CV %	Min	Max	Mean	CV %
7	101.0	159.0	130.5	15.68	103.0	150.0	125.4	11.63	111.8	150.5	125.9	8.67	111.4	144.8	127.3	10.15
14	29.56	54.0	35.36	19.60	28.00	35.22	32.24	6.60	28.86	35.33	31.75	6.31	27.75	35.00	31.66	6.87
21	13.29	19.67	15.92	11.71	14.75	17.88	15.96	5.43	13.67	16.78	14.97	6.81	14.11	18.11	15.43	7.29
C. 5 ppt	($N_{7^\circ}=3$, $N_{14^\circ}=4$, $N_{21^\circ}=2$)				($N_{7^\circ}=4$, $N_{14^\circ}=5$, $N_{21^\circ}=0$)				($N_{7^\circ}=6$, $N_{14^\circ}=7$, $N_{21^\circ}=1$)				($N_{7^\circ}=4$, $N_{14^\circ}=8$, $N_{21^\circ}=2$)			
Temperature	Min	Max	Mean	CV %	Min	Max	Mean	CV %	Min	Max	Mean	CV %	Min	Max	Mean	CV %
7	47.0	158.0	113.3	41.71	52.0	150.5	109.8	37.82	97.0	162.0	132.0	17.73	101.0	165.0	138.4	22.79
14	30.5	37.0	34.17	9.74	31.60	60.0	41.06	23.89	31.00	43.33	36.99	10.91	34.00	47.00	39.19	15.01
21	15.33	19.0	17.17	15.12	-	-	-	-	23.00	23.00	23.00	0	13.00	36.50	21.67	59.57

Table S3. Intrapopulation variation (Minimum, Maximum, Mean, and percent Coefficient of Variation (CV)) in length at hatching (mean mm for 10 eggs for each female) among females (*N* numbers) from four newt populations under different salinity - temperature treatment combinations. Values missing for HT for 5ppt and 21˚C due to 100% egg mortality in this treatment.

Treatment	Population															
A. 0 ppt	SC (*N* = 11)				HT (*N* = 12)				HF (*N* = 11)				HUF (*N* = 11)			
Temperature	Min	Max	Mean	CV %	Min	Max	Mean	CV %	Min	Max	Mean	CV %	Min	Max	Mean	CV %
7	6.70	9.57	8.97	9.14	9.28	10.12	9.64	2.61	9.52	10.43	9.99	3.27	9.35	10.34	9.77	3.08
14	8.77	12.76	9.61	11.64	8.98	10.82	9.94	4.94	9.06	10.94	10.34	5.32	9.70	10.48	10.12	2.72
21	8.54	9.72	9.23	4.27	9.31	10.01	9.72	1.96	9.52	10.40	9.97	2.33	9.14	10.15	9.74	2.65
B. 2 ppt	($N_{7°}$=8, $N_{14,21°}$=11)				(*N* = 12)				(*N* = 11)				(*N* = 11)			
Temperature	Min	Max	Mean	CV %	Min	Max	Mean	CV %	Min	Max	Mean	CV %	Min	Max	Mean	CV %
7	7.25	11.0	8.97	14.23	6.60	9.86	8.77	11.22	8.35	10.65	9.67	6.09	8.53	10.70	9.42	6.16
14	7.80	9.90	9.01	7.28	8.90	13.01	9.67	11.58	8.89	10.13	9.74	4.54	8.96	10.00	9.61	3.76
21	7.72	9.34	8.35	5.30	8.02	10.00	9.00	7.21	8.60	10.25	9.46	4.90	8.60	9.85	9.36	4.91
C. 5 ppt	($N_{7°}$=3, $N_{14°}$=4, $N_{21°}$=2)				($N_{7°}$=4, $N_{14°}$=5, $N_{21°}$=0)				($N_{7°}$=6, $N_{14°}$=7, $N_{21°}$=1)				($N_{7°}$=4, $N_{14°}$=8, $N_{21°}$=2)			
Temperature	Min	Max	Mean	CV %	Min	Max	Mean	CV %	Min	Max	Mean	CV %	Min	Max	Mean	CV %
7	5.00	6.50	5.70	13.25	3.50	8.40	5.41	43.91	5.70	9.00	7.42	16.74	4.50	7.55	5.96	22.47
14	5.70	8.65	6.91	18.74	4.90	8.84	16.56	21.81	5.10	8.03	6.50	16.44	4.90	8.93	6.72	21.56
21	7.83	9.30	8.57	12.11	-	-	-	-	7.00	7.00	7.00	0.00	3.80	10.10	6.95	64.10

Table S4. Intrapopulation variation (Minimum, Maximum, Mean, and percent Coefficient of Variation (CV)) in developmental stage at hatching (mean stage for 10 eggs for each female) among females (*N* numbers) from four newt populations under different salinity - temperature treatment combinations. Values missing for HT for 5ppt and 21˚C due to 100% egg mortality in this treatment.

Treatment	Population															
A. 0 ppt	SC (N = 11)				HT (N = 12)				HF (N = 11)				HUF (N = 11)			
Temperature	Min	Max	Mean	CV %	Min	Max	Mean	CV %	Min	Max	Mean	CV %	Min	Max	Mean	CV %
7	38.00	40.13	39.24	1.38	39.06	40.00	39.45	0.75	38.90	40.25	39.49	1.01	38.92	40.00	39.50	0.82
14	39.33	41.30	40.31	1.46	39.50	42.22	41.11	1.93	40.50	41.89	41.13	1.20	40.28	42.00	41.31	1.22
21	39.43	41.00	40.30	0.91	40.61	41.83	41.18	1.01	40.50	41.39	41.06	0.70	39.94	41.44	40.86	1.08
B. 2 ppt	($N_{7°}$=8, $N_{14,21°}$=11)				(N = 12)				(N = 11)				(N = 11)			
Temperature	Min	Max	Mean	CV %	Min	Max	Mean	CV %	Min	Max	Mean	CV %	Min	Max	Mean	CV %
7	38.13	41.00	39.76	2.20	38.00	40.93	39.71	2.60	38.75	41.50	40.25	2.22	38.90	41.75	40.15	2.25
14	39.11	43.00	40.40	3.04	37.40	41.94	40.00	2.88	39.29	41.50	40.24	1.81	38.88	42.00	40.51	2.55
21	39.07	40.89	40.08	1.49	39.67	41.67	40.58	1.44	39.17	41.67	40.50	1.70	39.39	41.38	40.75	1.36
C. 5 ppt	($N_{7°}$=3, $N_{14°}$=4, $N_{21°}$=2)				($N_{7°}$=4, $N_{14°}$=5, $N_{21°}$=0)				($N_{7°}$=6, $N_{14°}$=7, $N_{21°}$=1)				($N_{7°}$=4, $N_{14°}$=8, $N_{21°}$=0)			
Temperature	Min	Max	Mean	CV %	Min	Max	Mean	CV %	Min	Max	Mean	CV %	Min	Max	Mean	CV %
7	35.00	37.00	36.17	2.88	30.00	41.25	36.44	13.17	36.00	42.00	38.54	5.16	35.00	42.00	39.63	7.94
14	38.00	41.00	39.63	3.47	35.00	42.50	39.30	6.89	37.25	40.83	38.75	2.94	35.00	41.50	38.67	6.03
21	39.50	42.00	40.75	4.34	-	-	-	-	42.00	42.00	42.00	0.00	-	-	-	-

Table S5. Intrapopulation variation (Minimum, Maximum, Mean) in percent of hatchlings with developmental deformities (mean percent for 10 eggs for each female) among females (*N* numbers) from four newt populations under different salinity - temperature treatment combinations. Values missing for HT for 5ppt and 21˚C due to 100% egg mortality in this treatment.

Treatment	Population											
A. 0 ppt	SC ($N = 11$)			HT ($N = 12$)			HF ($N = 11$)			HUF ($N = 11$)		
Temperature	Min	Max	Mean	Min	Max	Mean	Min	Max	Mean	Min	Max	Mean
7	0.00	100.0	39.75	0.00	44.44	13.31	0.00	30.00	10.61	0.00	57.14	19.66
14	0.00	14.29	2.31	0.00	60.00	5.00	0.00	11.11	2.020	0.00	11.11	1.01
21	0.00	14.29	2.60	0.00	22.22	4.894	0.00	0.00	0.00	0.00	12.50	5.30
B. 2 ppt	($N_{7°}$=8, $N_{14,21°}$=11)			($N = 12$)			($N = 11$)			($N = 11$)		
Temperature	Min	Max	Mean	Min	Max	Mean	Min	Max	Mean	Min	Max	Mean
7	50.0	100	87.5	20.00	100.0	67.48	28.57	100	68.07	33.33	100	82.77
14	0.00	100	44.07	0.00	57.14	16.78	0.00	25.00	7.87	0.00	100	28.14
21	0.00	100	62.01	0.00	100.0	47.56	0.00	44.44	13.59	0.00	100	36.94
C. 5 ppt	($N_{7°}$=3, $N_{14°}$=4, $N_{21°}$=2)			($N_{7°}$=4, $N_{14°}$=5, $N_{21°}$=0)			($N_{7°}$=6, $N_{14°}$=7, $N_{21°}$=1)			($N_{7°}$=4, $N_{14°}$=8, $N_{21°}$=2)		
Temperature	Min	Max	Mean	Min	Max	Mean	Min	Max	Mean	Min	Max	Mean
7	100	100	100	100	100	100	100	100	100	100	100	100
14	50.0	100	87.50	88.00	100	97.60	100	100	100	66.67	100	95.83
21	0.00	33.33	16.67	-	-	-	100	100	100	0	100	50.00

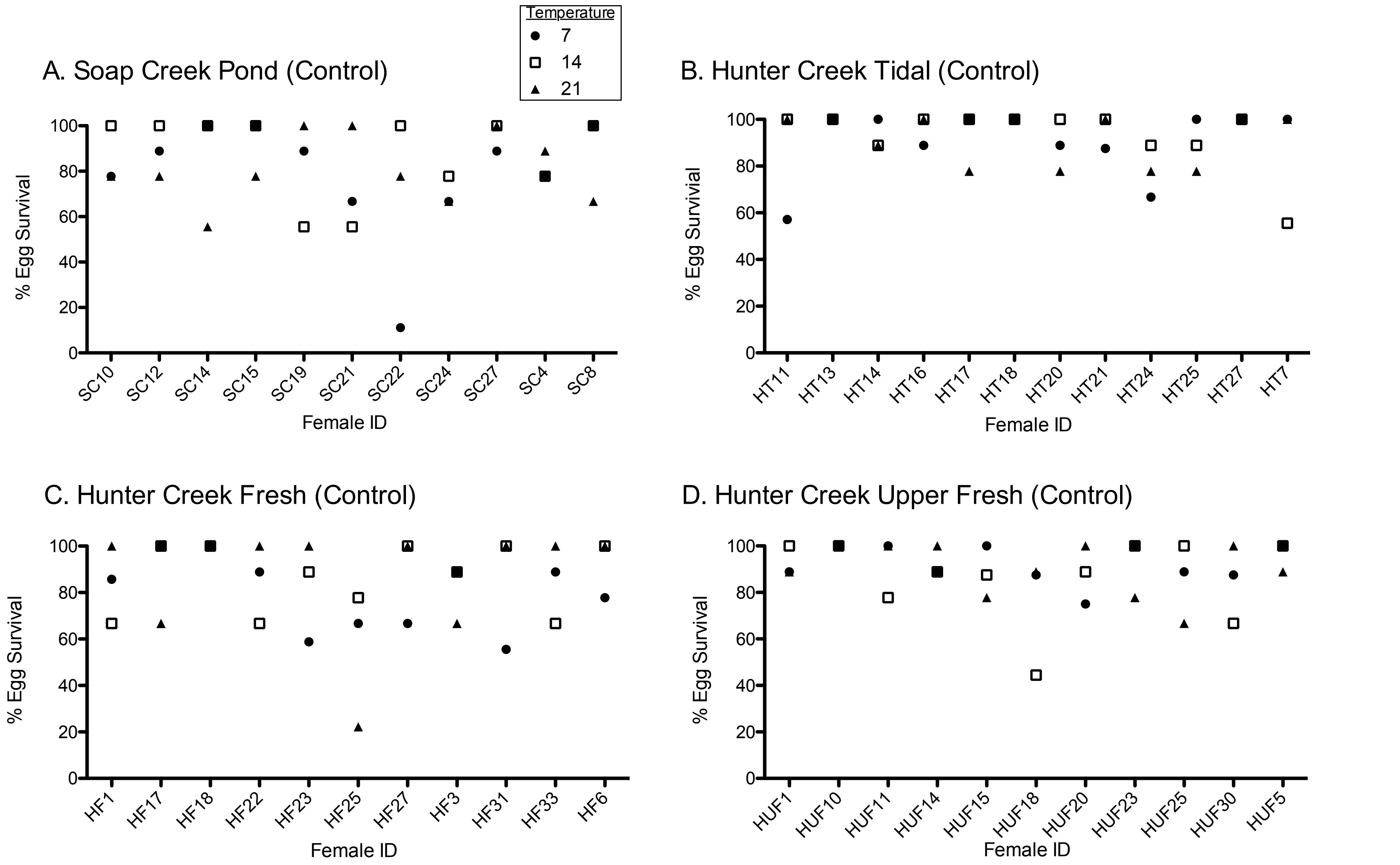

Figure S1. Interfamily variation in percent survival of eggs from 11-12 different female newts (*Taricha granulosa*) from each of four different populations in Control (0.2 ppt salinity) in different temperature treatments.

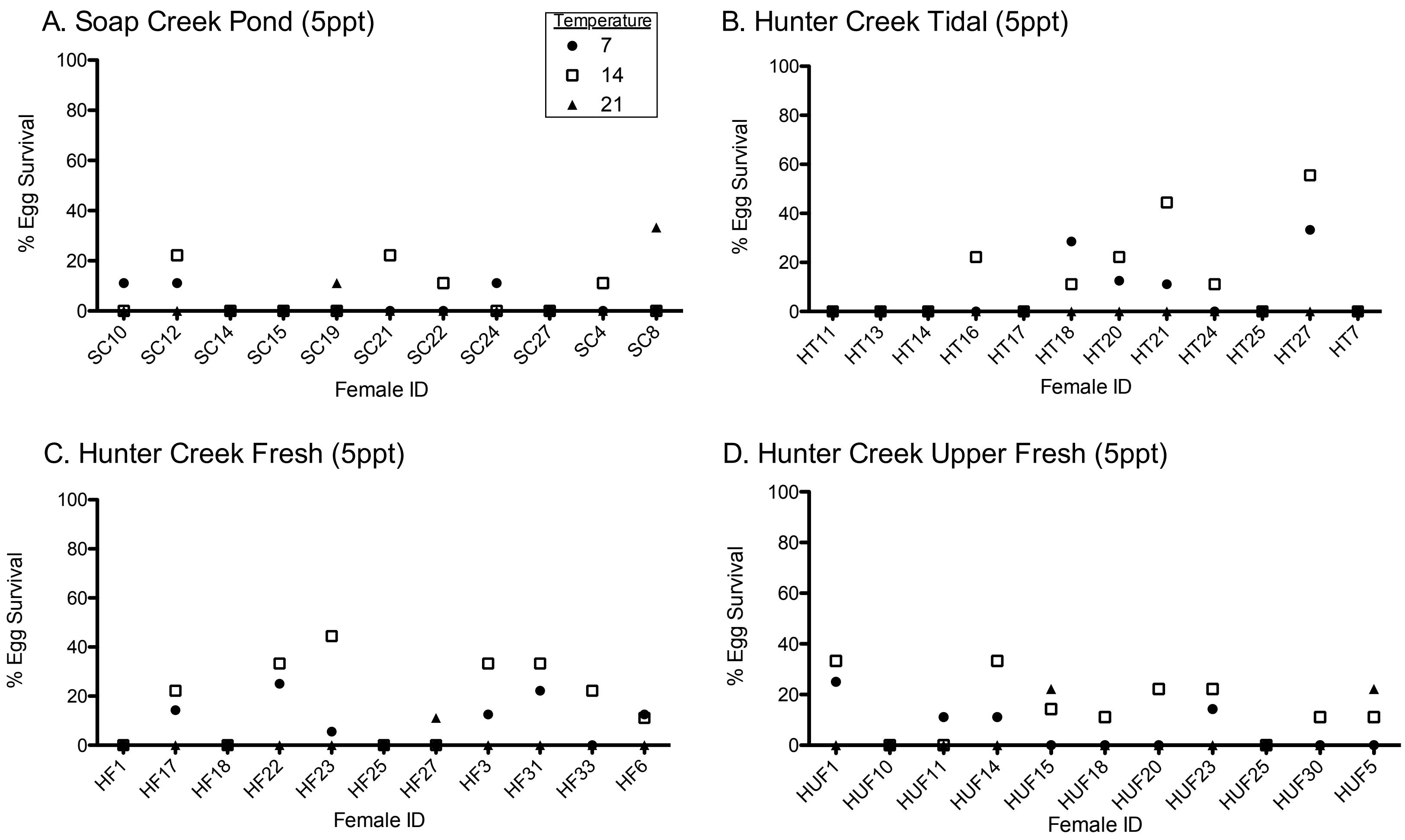

Figure S2. Interfamily variation in percent survival of eggs from 11-12 different female newts (*Taricha granulosa*) from each of four different populations in 5 ppt salinity in different temperature treatments. Missing values arise from some females experiencing 100% egg mortality in a specific salinity – temperature combination.

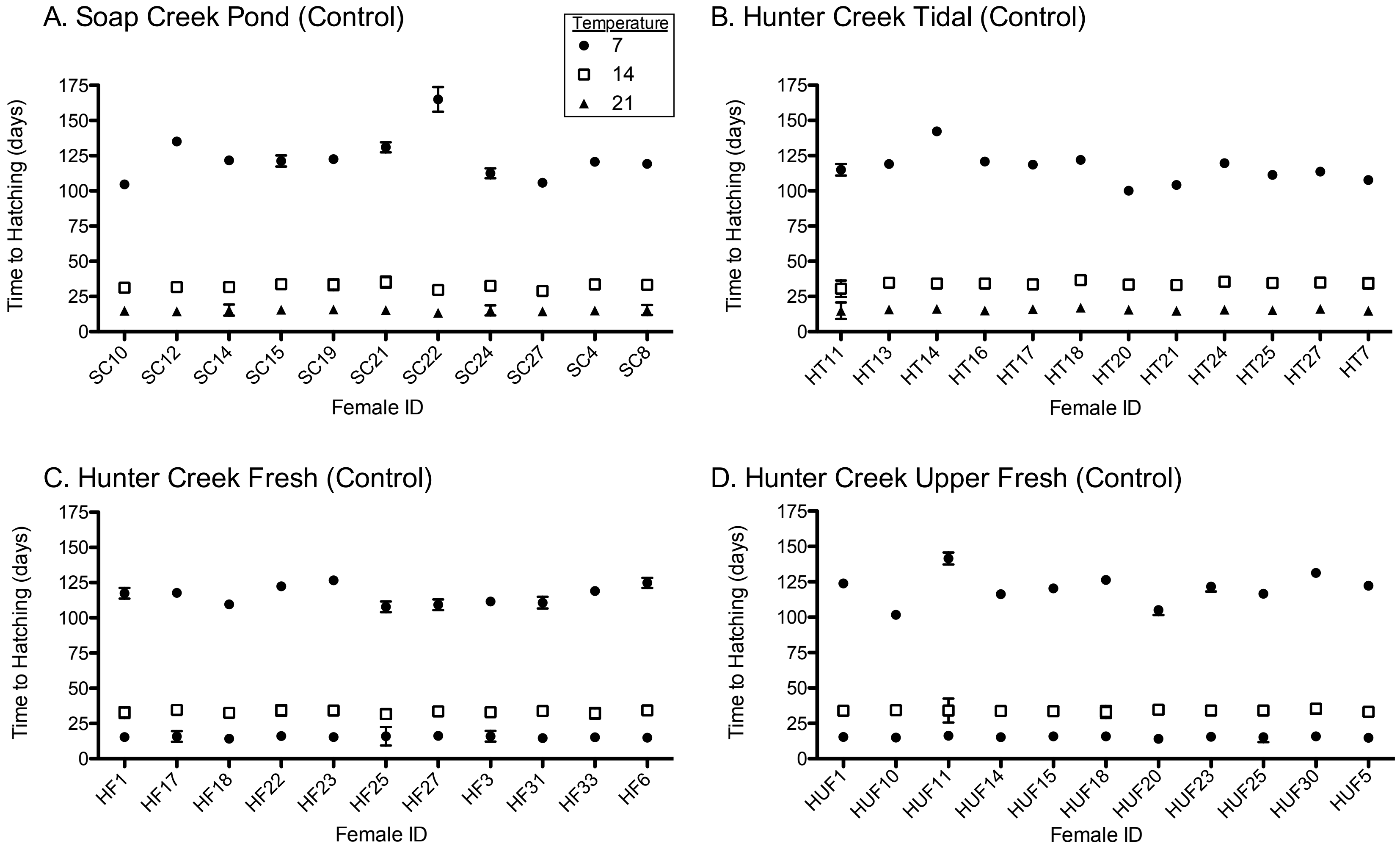

Figure S3. Interfamily variation in time to hatching from 11-12 different female newts (*Taricha granulosa*) from each of four different populations in Control (0.2 ppt) salinity in different temperature treatments.

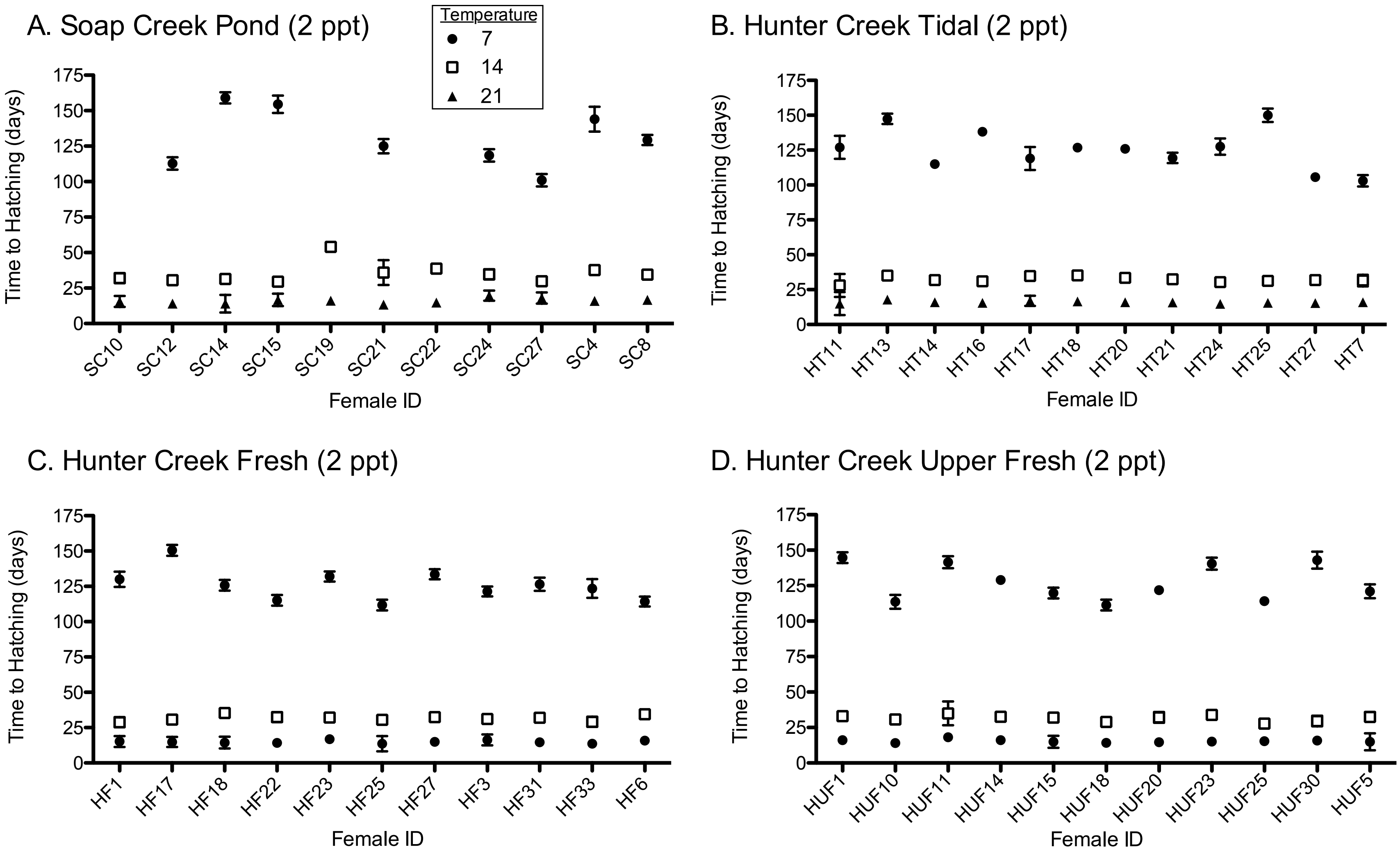

Figure S4. Interfamily variation in time to hatching from 11-12 different female newts (*Taricha granulosa*) from each of four different populations in 2 ppt salinity in different temperature treatments. Missing values arise from some females experiencing 100% egg mortality in a specific salinity – temperature combination.

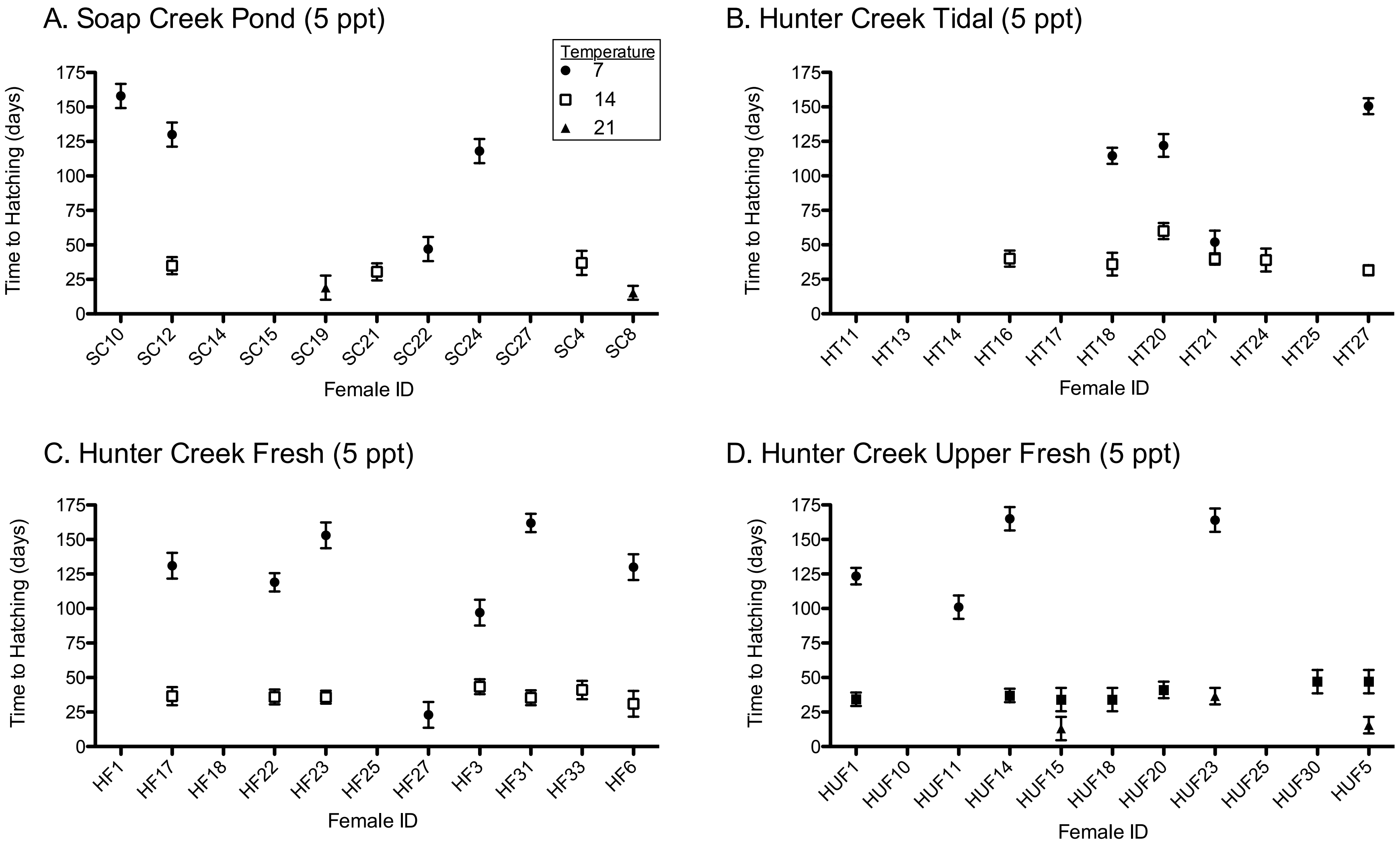

Figure S5. Interfamily variation in time to hatching from 11-12 different female newts (*Taricha granulosa*) from each of four different populations in 5 ppt salinity in different temperature treatments. Missing values arise from some females experiencing 100% egg mortality in a specific salinity – temperature combination.

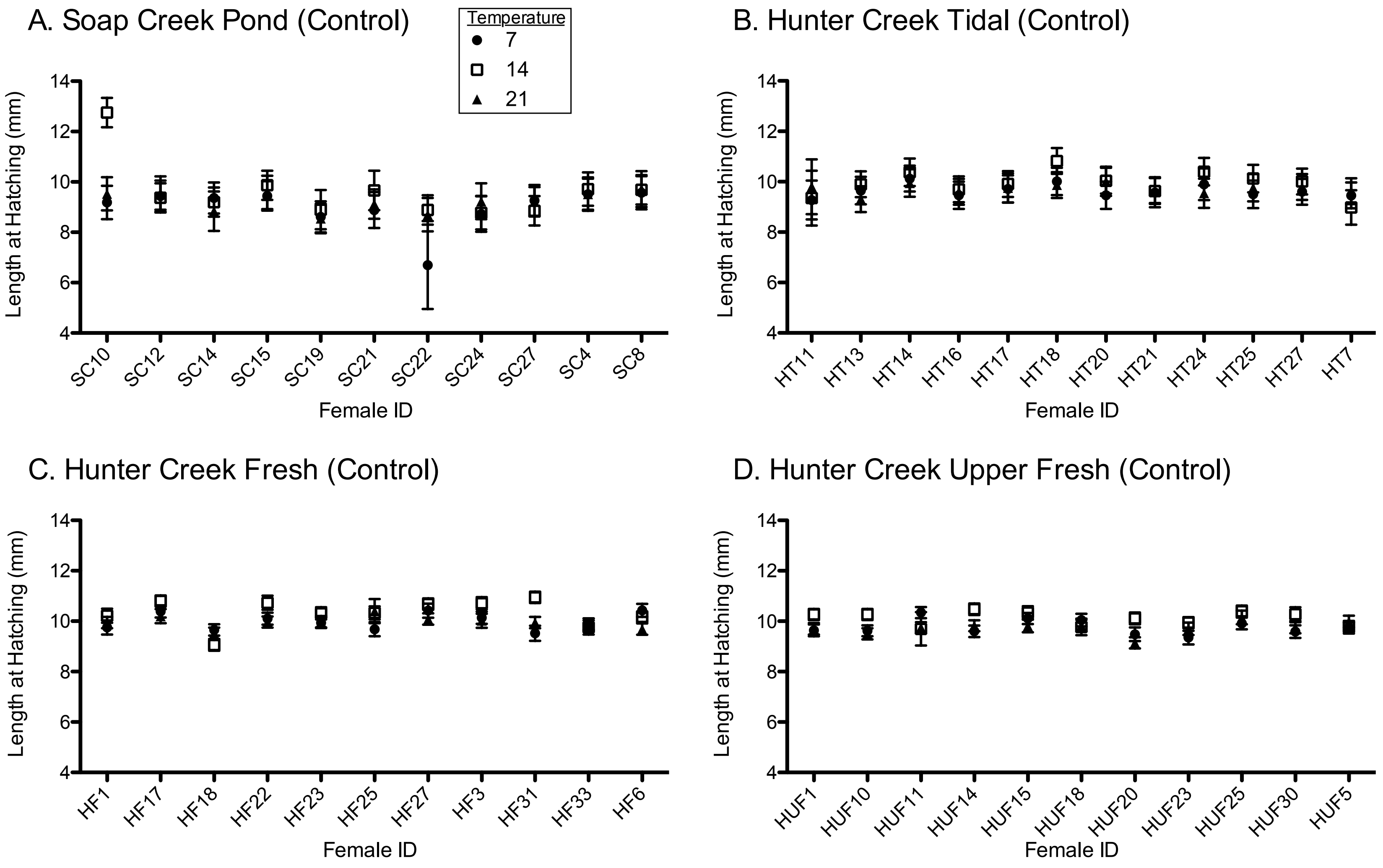

Figure S6. Interfamily variation in length at hatching from 11-12 different female newts (*Taricha granulosa*) from each of four different populations in Control (0.2 ppt) salinity in different temperature treatments.

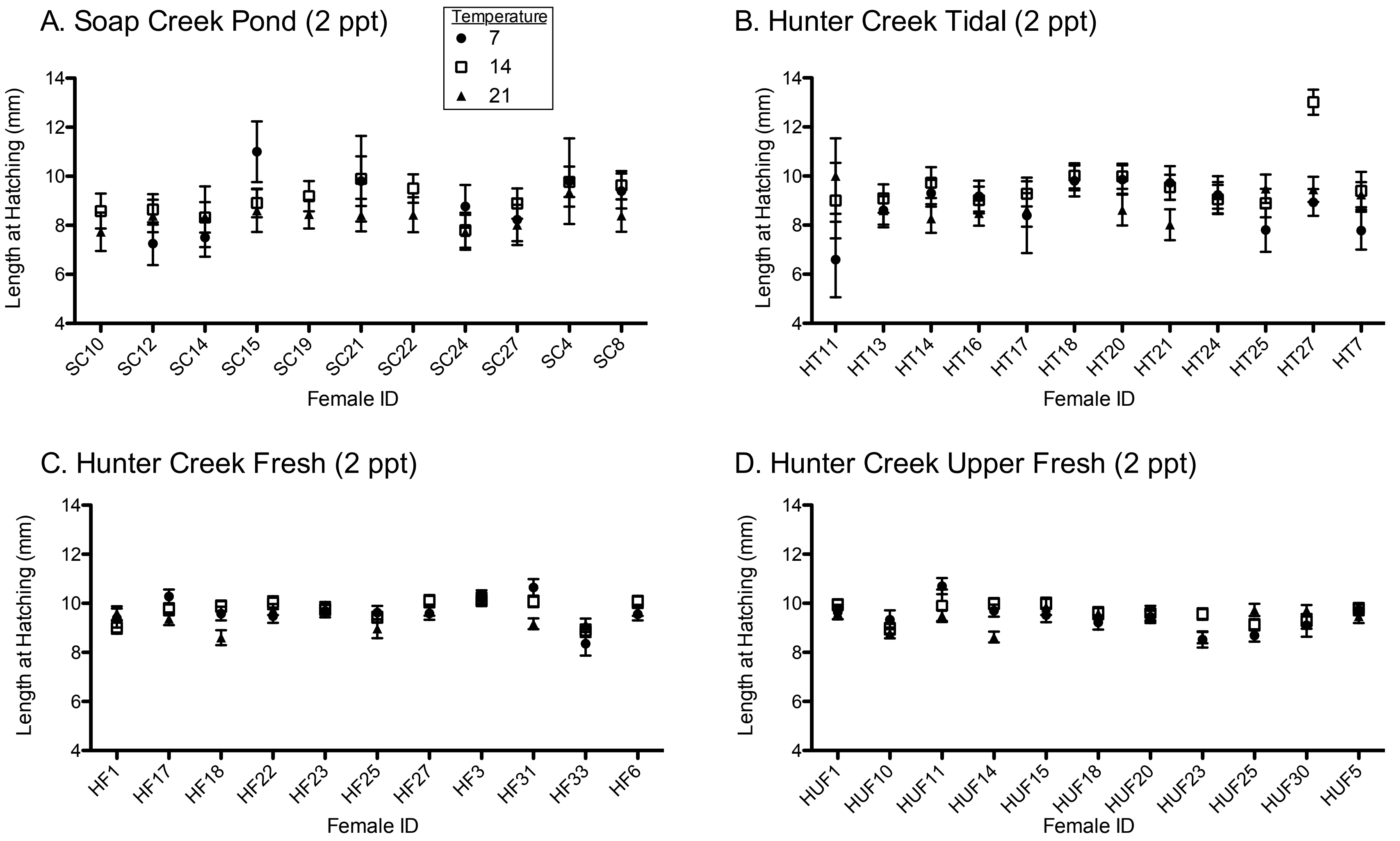

Figure S7. Interfamily variation in length at hatching from 11-12 different female newts (*Taricha granulosa*) from each of four different populations in 2 ppt salinity in different temperature treatments. Missing values arise from some females experiencing 100% egg mortality in a specific salinity – temperature combination.

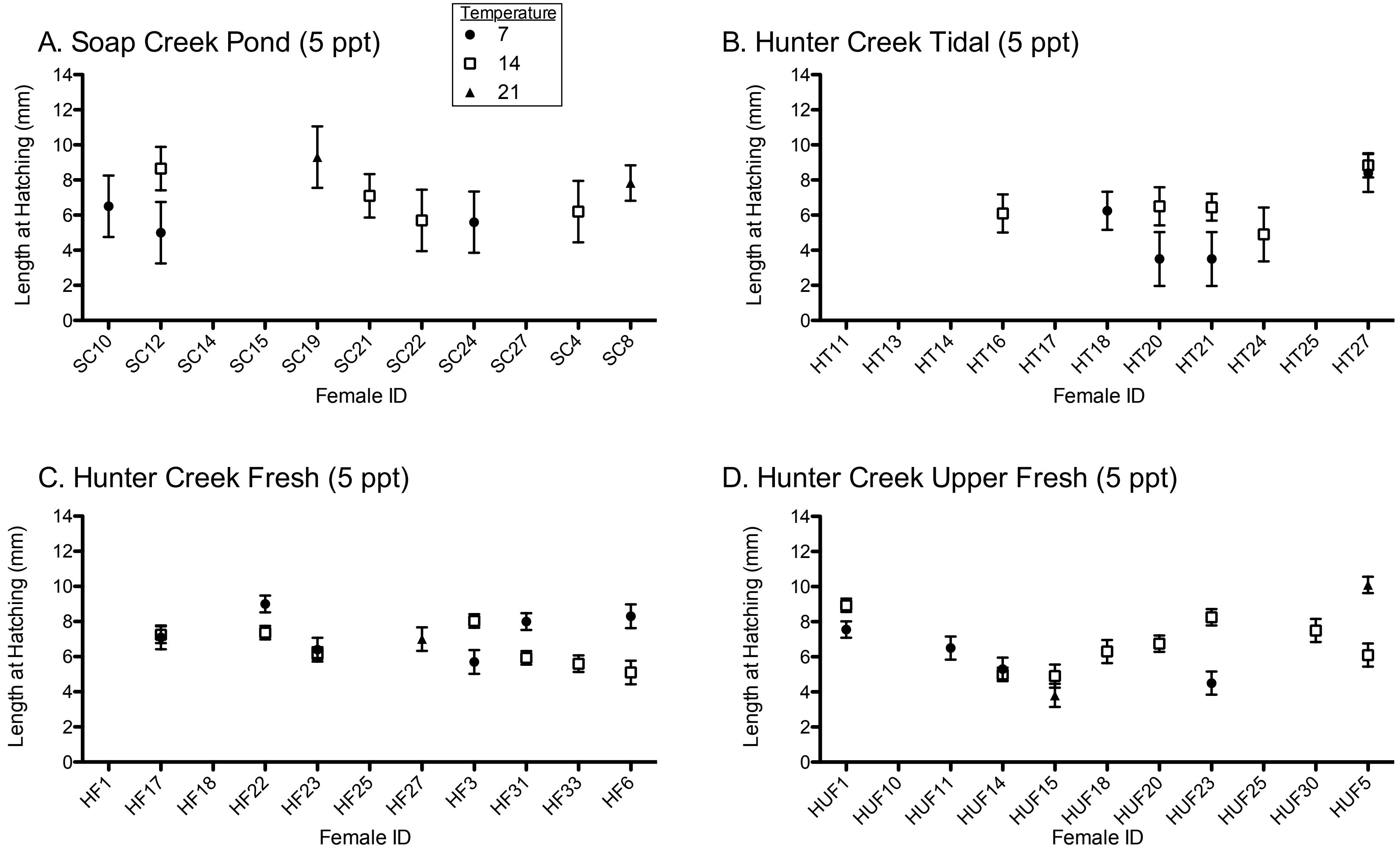

Figure S8. Interfamily variation in length at hatching from 11-12 different female newts (*Taricha granulosa*) from each of four different populations in 5 ppt salinity in different temperature treatments. Missing values arise from some females experiencing 100% egg mortality in a specific salinity – temperature combination.

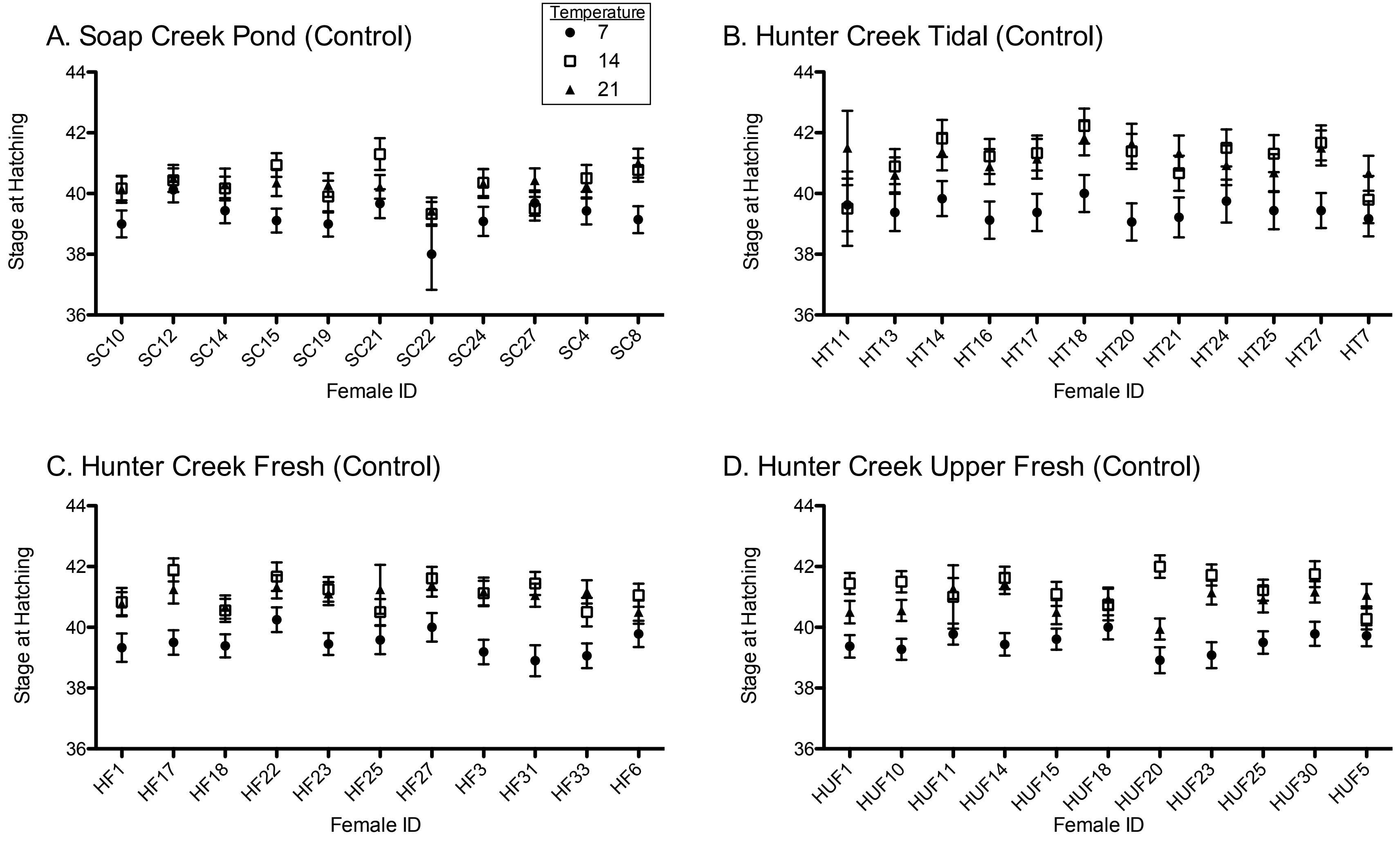

Figure S9. Interfamily variation in developmental stage at hatching from 11-12 different female newts (*Taricha granulosa*) from each of four different populations in Control (0.2 ppt) salinity in different temperature treatments.

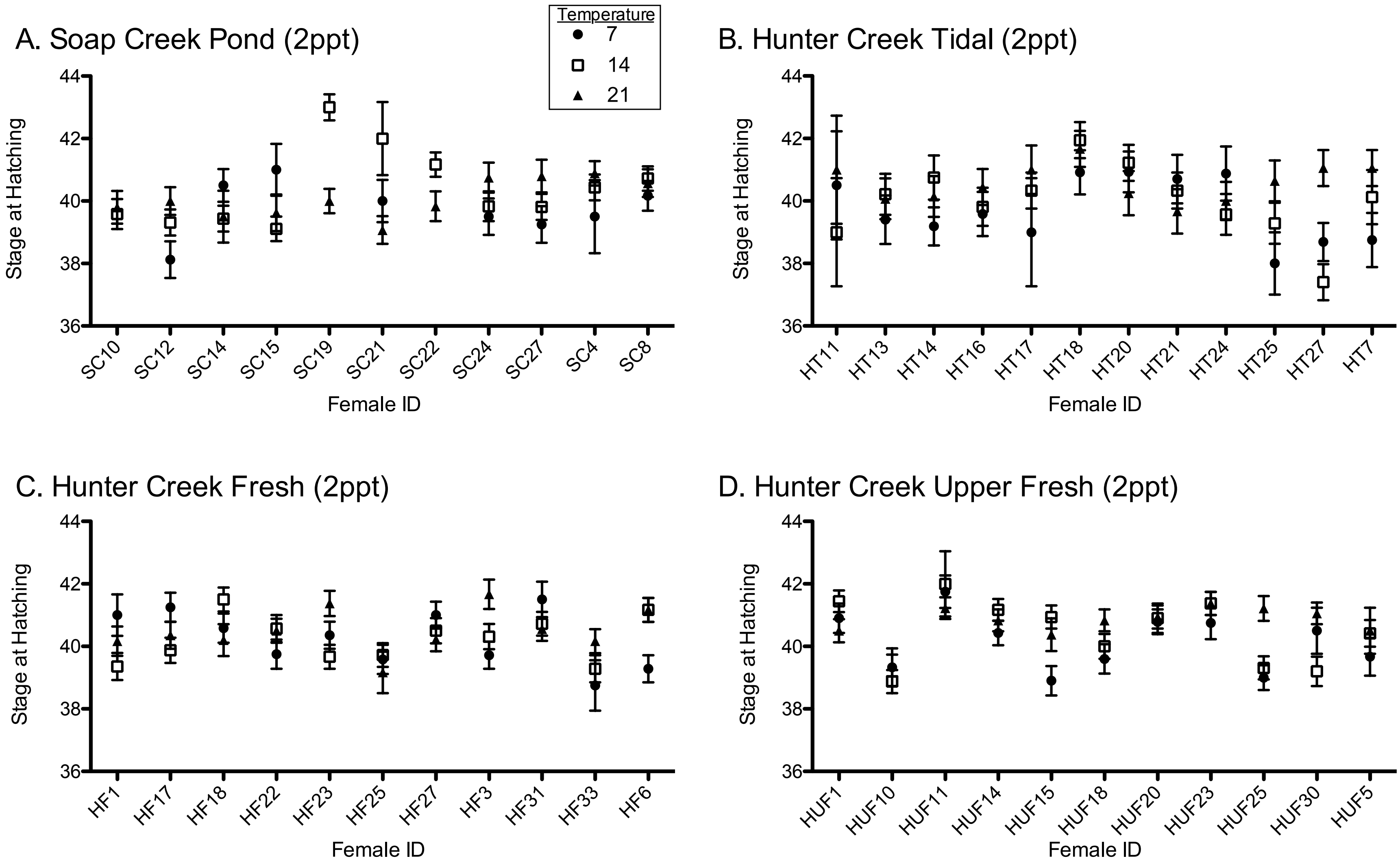

Figure S10. Interfamily variation in developmental stage at hatching from 11-12 different female newts (*Taricha granulosa*) from each of four different populations in 2 ppt salinity in different temperature treatments. Missing values arise from some females experiencing 100% egg mortality in a specific salinity – temperature combination.

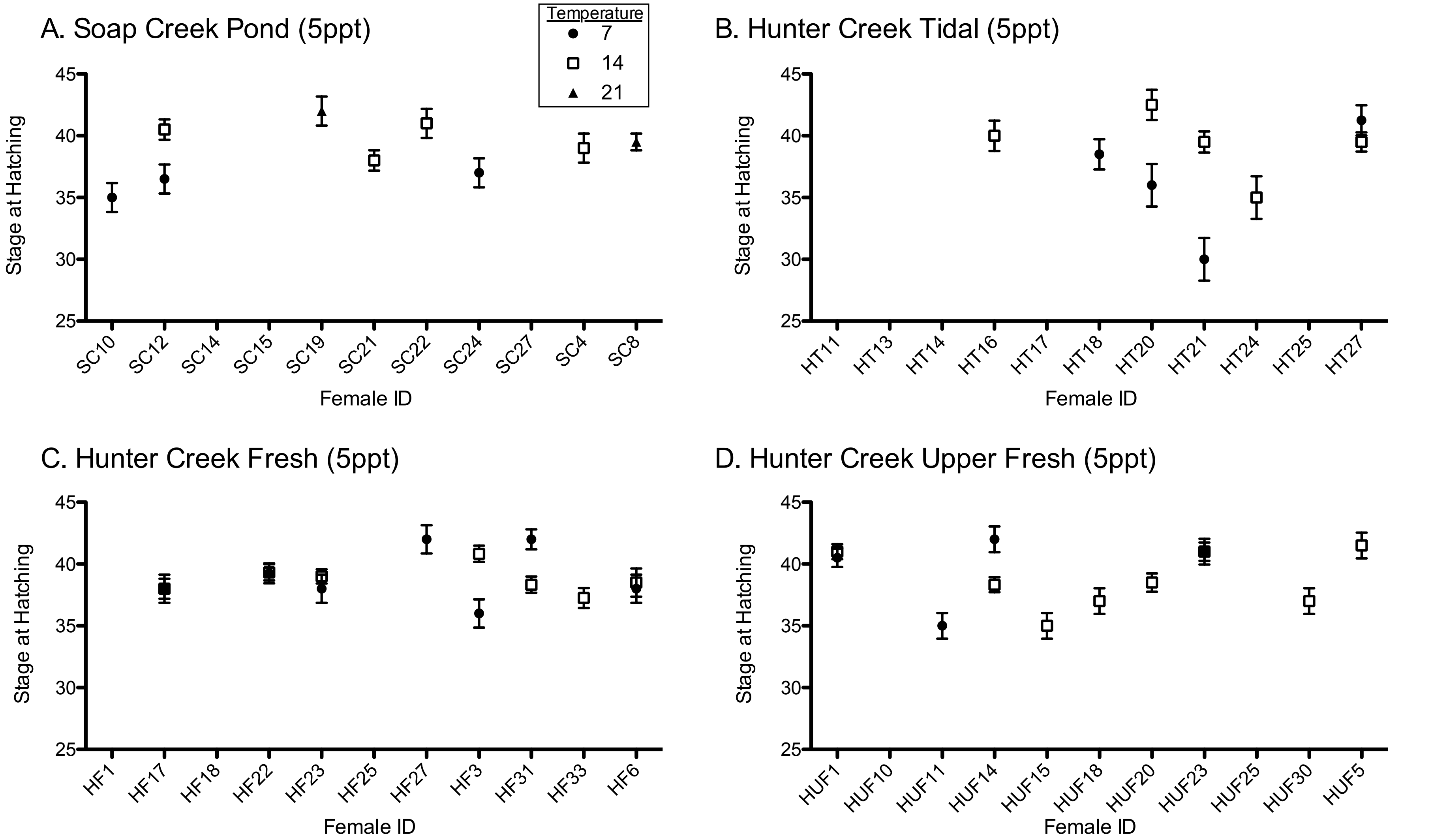

Figure S11. Interfamily variation in developmental stage at hatching from 11-12 different female newts (*Taricha granulosa*) from each of four different populations in 5 ppt salinity in different temperature treatments. Missing values arise from some females experiencing 100% egg mortality in a specific salinity – temperature combination.

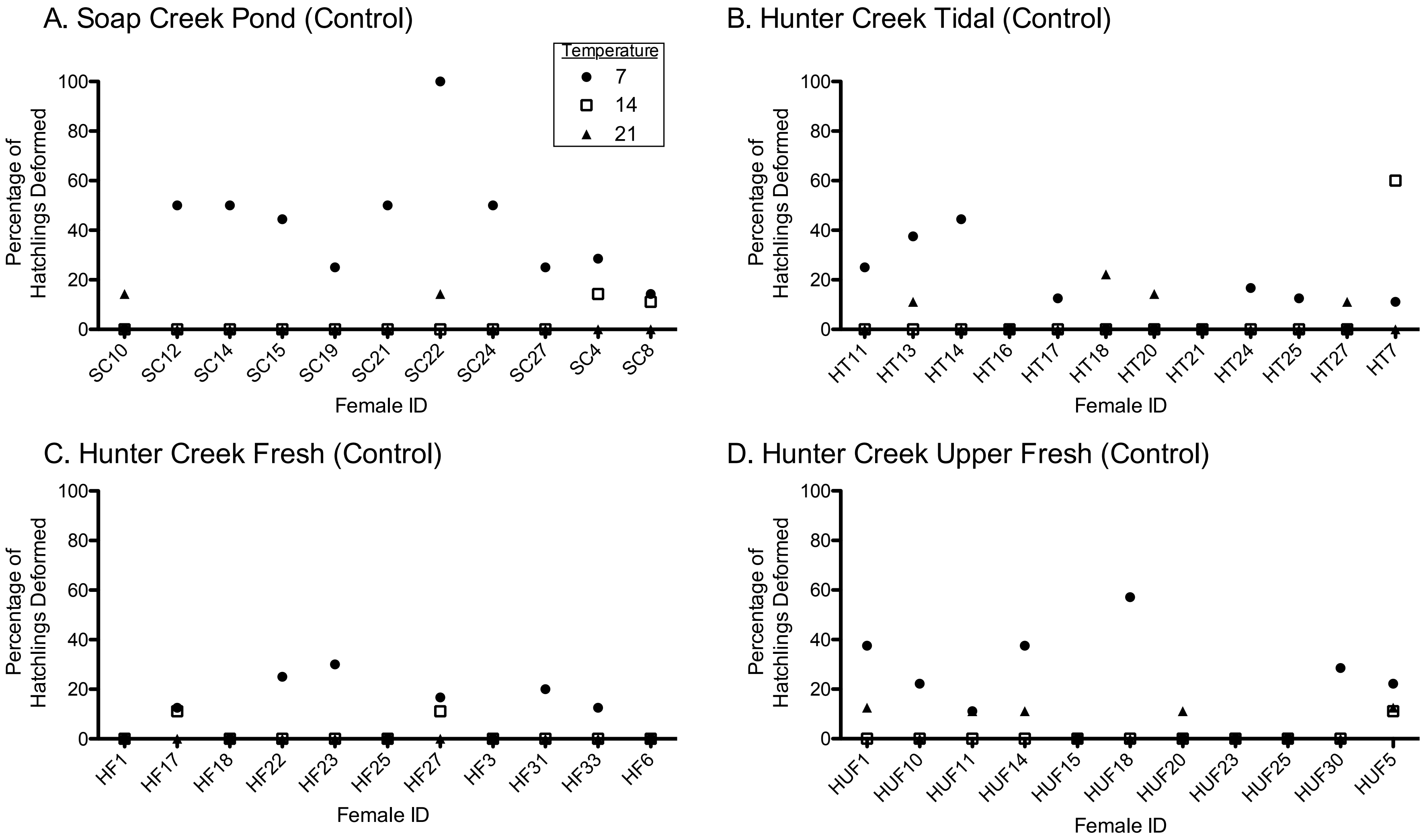

Figure S12. Interfamily variation in percentage of hatchlings with developmental deformities from 11-12 different female newts (*Taricha granulosa*) from each of four different populations in Control (0.2 ppt) salinity in different temperature treatments.

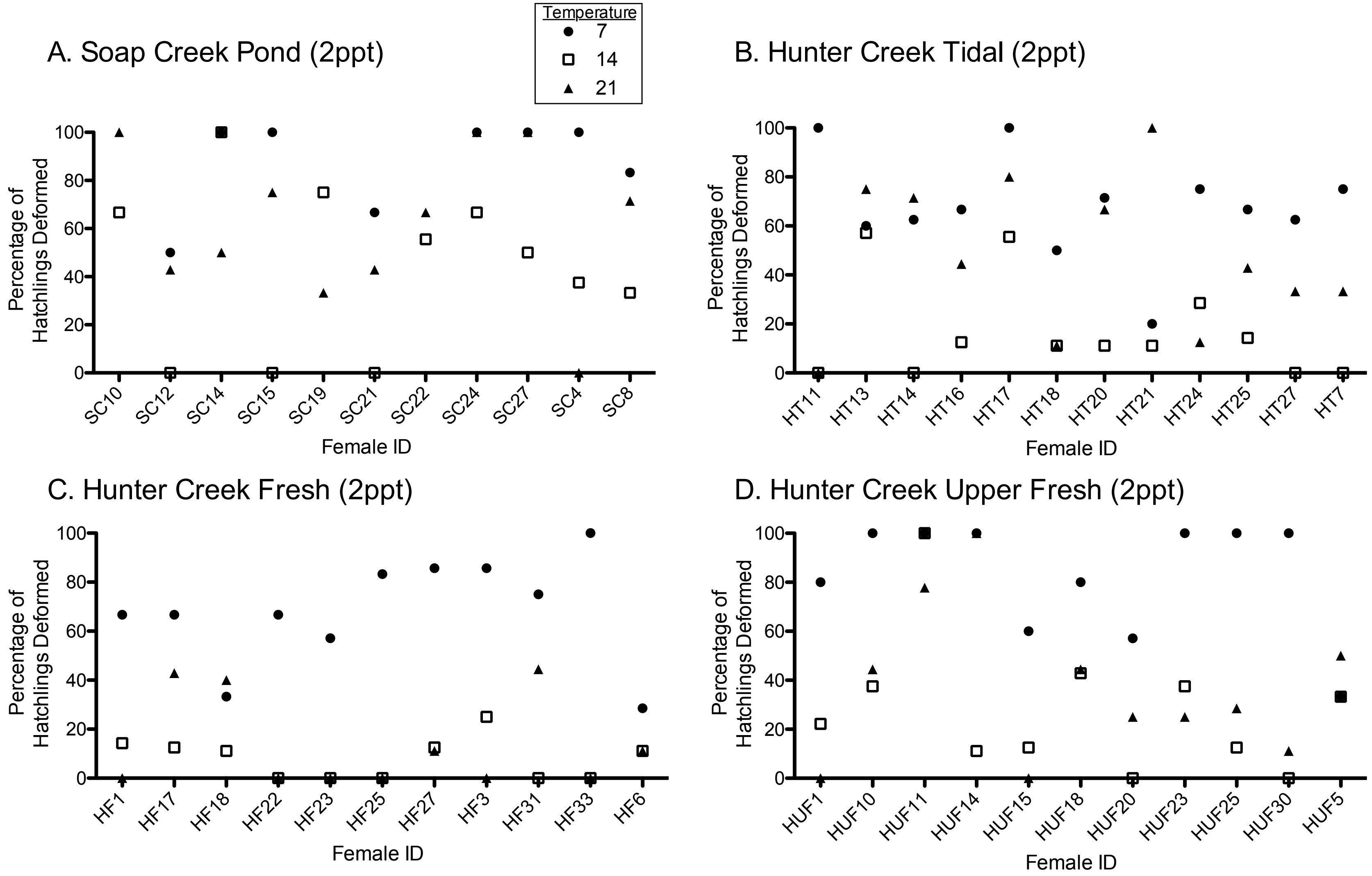

Figure S13. Interfamily variation in percentage of hatchlings with developmental deformities from 11-12 different female newts (*Taricha granulosa*) from each of four different populations in 2 ppt salinity in different temperature treatments. Missing values arise from some females experiencing 100% egg mortality in a specific salinity – temperature combination.

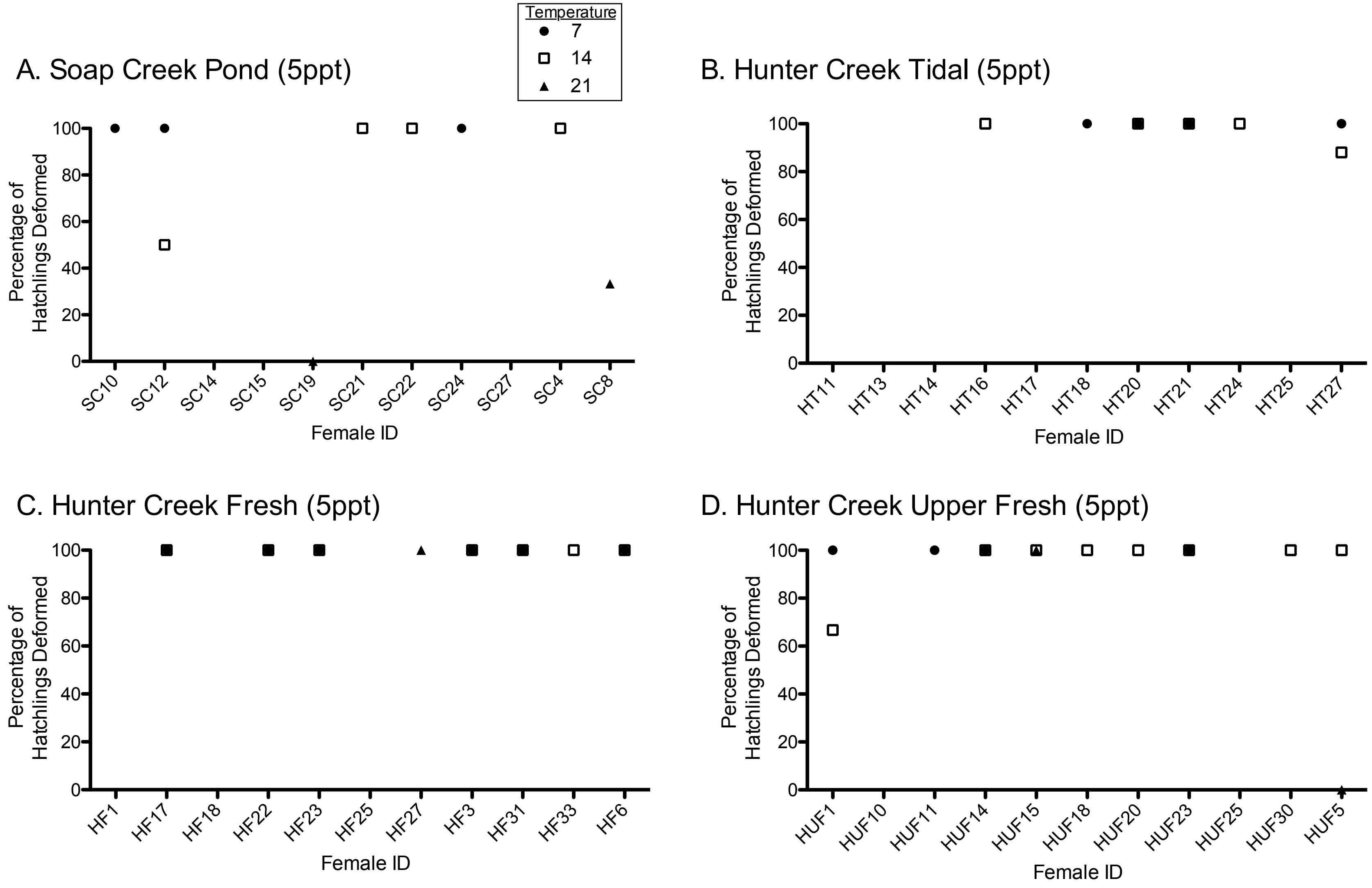

Figure S14. Interfamily variation in percentage of hatchlings with developmental deformities from 11-12 different female newts (*Taricha granulosa*) from each of four different populations in 5 ppt salinity in different temperature treatments. Missing values arise from some females experiencing 100% egg mortality in a specific salinity – temperature combination.
